# Expression of miRNA and Occurrence of Distant Metastases in Patients with Hürthle Cell Carcinoma

**DOI:** 10.1155/2016/8945247

**Published:** 2016-07-28

**Authors:** Rok Petric, Barbara Gazic, Katja Goricar, Vita Dolzan, Radan Dzodic, Nikola Besic

**Affiliations:** ^1^Department of Surgical Oncology, Institute of Oncology, Zaloska 2, SL-1000, Ljubljana, Slovenia; ^2^Department of Pathology, Institute of Oncology, Zaloska 2, SL-1000, Ljubljana, Slovenia; ^3^Pharmacogenetics Laboratory, Institute of Biochemistry, Faculty of Medicine, University of Ljubljana, SL-1000, Ljubljana, Slovenia; ^4^Department of Surgical Oncology, Institute of Oncology and Radiology of Serbia, Pasterova 2, 11000 Belgrade, Serbia

## Abstract

*Background*. Hürthle cell thyroid carcinoma (HCTC) is a rare type of thyroid carcinoma. In the present study, we investigated whether the expression of miRNAs of interest is associated with the occurrence of metastases in patients with HCTC.* Materials and Methods*. In 39 patients with HCTC (22 with nonmetastatic and 17 with regional or distant metastatic disease), the expression levels of six miRNAs (miR-138, miR-183, miR-221, miR-222, miR-768-3p, and miR-885-5p) and U6 snRNA as endogenous control were determined in FFPE samples of primary tumor and normal thyroid tissue using TaqMan miRNA assays.* Results*. In patients with HCTC, miR-138 and miR-768-3p were downregulated in tumor samples compared to normal tissue (*p* = 0.013 and *p* = 0.010, resp.). These two miRNAs were also significantly downregulated in tumor samples of patients with metastatic disease (*p* = 0.030 and *p* = 0.048, resp.) but not in patients with nonmetastatic disease (*p* = 0.249 and *p* = 0.101, resp.). In patients with nonmetastatic disease, miR-221 and miR-885-5p were slightly, albeit significantly, upregulated in tumorous compared to normal tissue (*p* = 0.042 and *p* = 0.027, resp.).* Conclusion*. Expression of miRNA (miR-183, miR-221, and miR-885-5p) in tumor tissue is associated with the occurrence of distant metastases in patients with HCTC.

## 1. Introduction

Hürthle cell carcinoma of the thyroid gland (HCTC) is a rare type of differentiated thyroid cancer, which accounts for around 3% of all thyroid malignancies [1, 2]. There are only a limited number of publications in the literature about prognostic factors in patients with HCTC [1–15].

According to the World Health Organization classification, HCTC is considered an oxyphilic variant of follicular thyroid carcinoma [16], but genomic dissection of HCTC revealed a unique class of thyroid malignancy distinct from papillary and follicular carcinoma [1, 4]. Dettmer et al. found out that miRNA (miR-885-5p) was found to be strongly upregulated (40-fold) in HCTC but not in classical follicular thyroid carcinomas, follicular adenomas, or hyperplastic nodules [17]. So, miR-885-5p may serve as a diagnostic marker for HCTC. Furthermore, miR-23b was associated with tumor relapses, while miR-150 was associated with tumor specific death in poorly differentiated HCTC [18]. In the present study, we investigated if miRNAs that were previously reported to be differentially expressed in HCTC are associated with the occurrence of metastases in patients with HCTC.

## 2. Materials and Methods

### 2.1. Patients

Altogether, 108 patients with HCTC were treated at our institute from 1972 to 2011. Among these, 32 patients (19 females, 13 males; median age: 64.5 years) had either an initially proven metastatic disease (*N* = 12) or metastatic disease that occurred after initial treatment (*N* = 20) [19]. All the histological slides taken from our patients with HCTC were examined by a pathologist with experience in thyroid pathomorphology (BG). HCTC was diagnosed based on the histological criteria defined by Rosai [20], LiVolsi and Baloch [21], and Nikiforov et al. [22]. All follicular carcinomas in which at least 75% of the follicular cells had oncocytic characteristics were included in the study group. The identification of oncocytic characteristics was based on the presence of follicular cells with abundant acidophilic, granular cytoplasm with small or medium sized, round nuclei with vesicular chromatin and prominent nucleoli. The diagnosis of malignancy was based on histologic evidence of vascular and/or transcapsular invasion [20–22] or presence of nodal or distant metastasis. All patients with Hürthle cell neoplasms with cells containing typical nuclear features of papillary carcinoma were excluded from our present study and were the subject of one of our previous studies [23]. Seven patients had poorly differentiated HCTC according to older criteria [20, 21].

In the whole patient cohort, 39 patients with HCTC (22 with nonmetastatic and 17 with regional or distant metastases) fulfilled the histopathological criteria and also had available clinical data and a sufficient amount of formalin fixed paraffin embedded (FFPE) samples to be included in the present study. Only two of 39 cases had HCTC which fulfilled diagnostic criteria for poorly differentiated thyroid carcinoma (Turin criteria): the presence of solid/trabecular/insular growth pattern, absence of conventional nuclear features of papillary carcinoma, and the presence of at least one of the following features: convoluted nuclei, mitotic activity ≥3/10 high-power fields, or tumor necrosis [24].

We reviewed the medical charts and collected data on patients' gender, age, clinical and histopathological factors, tumor stage, and recurrence. The tumor stage and presence of regional and/or distant metastases were assessed by the TNM clinical classification according to the UICC criteria from 2009 [25]. All patients with HCTC were monitored regularly at least once per year at our institute for possible recurrent or metastatic disease as already reported by our group [15, 19].

The Medical Ethics Committee of the Republic Slovenia and The Protocol Review Board and Ethics Committee of the Institute of Oncology reviewed and approved the study, which was conducted in accordance with the ethical standards prescribed in the Declaration of Helsinki. For retrospective studies, written consent is not necessary according to national regulations. The need for consent was waived by the Institutional Review Board and Ethics Committee of the Institute of Oncology Ljubljana.

#### 2.1.1. DNA Isolation and Genotyping

Hematoxylin and eosin (H&E) stained slides from FFPE samples were reviewed by the pathologist, who confirmed the diagnosis and selected areas representative of both HCTC and normal tissue. Two to three cores (1 mm in diameter) of histologically confirmed HCTC and of normal tissue were obtained from each specimen. For all the patients, miRNA was extracted from FFPE samples of primary tumor and of normal thyroid tissue using Qiagen miRNeasy FFPE Kit (Quiagen, Hilden, Germany) according to the manufacturer's instructions. Quality of RNA and its size were assessed using the Agilent Small RNA kit (Agilent Technologies, California, USA). The expression of six miRNAs (miR-138, miR-183, miR-221, miR-222, miR-768-3p, and miR-885-5p) and U6 snRNA as endogenous control was determined using TaqMan MicroRNA Reverse Transcription Kit and specific TaqMan miRNA assays (TaqMan, Applied Biosystems, California, USA). Reactions for all miRNA assays and for all normal and tumor samples were performed in triplicate using the same amount of isolated RNA. Specific miRNAs, which were analyzed in our study, were selected based on data from the literature [17, 26–28].

#### 2.1.2. Statistical Analysis

Median and interquartile ranges were used to describe continuous variables, while frequencies were used to describe the distribution of categorical variables. Relative quantification was used to compare miRNA levels. The Wilcoxon test for paired samples was used to compare the relative expression levels of miRNA normalized to U6 snRNA expression in tumor and normal samples from each patient, while the Mann-Whitney* U* test was used to compare relative expression of miRNA calculated as the fold change of expression of miRNA of interest in tumor samples with the expression of miRNA of interest in normal samples (both normalized to U6 snRNA expression) among patients with and without metastases. All statistics were analyzed using IBM SPPS Statistics version 19.0 (IBM Corporation, Armonk, NY, USA). The level of statistical significance was set at 0.05.

## 3. Results

The present study included 39 patients with HCTC. Among them, 22 patients had a nonmetastatic and 17 a regional or distant metastatic disease. Four patients had regional or distant metastases at the time of diagnosis, while 13 patients had regional or distant recurrence. The patients' clinical and pathomorphological characteristics are given in Table 1.

Patients with a metastatic disease were 11 years older than patients with a nonmetastatic disease (*p* = 0.006) and had a significantly larger median size of their initial tumor (6.5 versus 3 cm) (*p* = 0.001). They also had a significantly higher percentage of tumors in stage pT3 or stage pT4 (*p* = 0.008). The extent of vascular invasion and transcapsular invasion and poorly differentiated morphology as assessed by Turin criteria were not associated with metastatic disease (Table 1).

The relative expression of miRNAs in normal and tumor samples of all patients, patients without metastases, and patients with metastases is presented in Tables 2, 3, and 4, respectively. A comparison of the relative expressions of miRNA among patients without metastases and patients with metastases is presented in Table 5.

In all patients with HCTC, miR-138 and miR-768-3p were downregulated in tumor samples compared to normal tissue (*p* = 0.013 and *p* = 0.010, resp.) (Table 2). These two miRNAs were also significantly downregulated in tumor samples compared to normal tissue from patients with a metastatic disease (*p* = 0.030 and *p* = 0.048, resp.) (Table 4) but not in patients with a nonmetastatic disease (*p* = 0.249 and *p* = 0.101, resp.) (Table 3). In patients with a nonmetastatic disease, miR-221 and miR-885-5p were slightly, albeit significantly, upregulated in tumorous compared to normal tissue (*p* = 0.042 and *p* = 0.027, resp.) (Table 3). When fold change of miRNA expression in tumor versus normal samples was compared between patients with and without a metastatic disease, significant downregulation of miR-183, miR-221, and miR-885-5p was observed in patients with a metastatic disease (*p* = 0.027, *p* = 0.019, and *p* = 0.024, resp.) (Table 5).

The expression of investigated miRNA in two patients with poorly differentiated tumors was similar to the expression in patients with well differentiated tumors. Comparing these two poorly differentiated tumors with the rest, relative expression of miR-138 was 0.14 (0.05–0.14) compared to 0.13 (0.01–1.09). For miR-221, relative expression was 0.96 (0.08–0.96) compared to 1.06 (0.28–9.10), for miR-222, 1.64 (0.10–1.64) compared to 0.99 (0.46–6.36), for miR-768-3p, 0.53 (0.08–0.53) compared to 0.62 (0.08–1.11), and for miR-885-5p, 0.51 (0.00–0.51) compared to 1.04 (0.28–5.39). Moreover, miRNA expression in patients with minimally invasive tumors did not differ significantly from miRNA expression in patients with widely invasive or poorly differentiated tumors (Table 6).

## 4. Discussion

In the present study, we investigated factors associated with the occurrence of distant metastases in patients with HCTC. A significant difference in age was observed between the metastatic group and nonmetastatic HCTC group, with patients in the HCTC group being 11 years older and having a significantly larger median initial tumor size (6.5 versus 3 cm) and higher pT stage. These findings are consistent with previous reports [15, 29, 30]. Extent of invasion or differentiation was not associated with metastatic disease in our present study.

MiRNAs can function as oncogenes or tumor suppressor genes by regulating the expression of target genes through loss or gain of miRNA functions [31]. Accounts of the usefulness of various miRNAs for diagnostic purposes in Hürthle cell neoplasms have been already published [17, 26], but to our knowledge, there are only very limited data in the literature about miRNAs and occurrence of metastases in patients with HCTC and our study is the first investigating miRNA expression only in HCTC using paired normal and tumor tissue, as well as investigating the association with metastatic disease.

An important finding of our study is that significantly reduced expression of miR-138 and miR-768-3p was observed when tumor samples were compared to normal tissue in all HCTC patients and also in patients with distant metastases. The same trend, though insignificant, was seen in patients without metastases. However, the relative expression of these two miRNAs was not significantly different in patients without metastases compared to patients with metastatic disease. Vriens et al. [26] compared miRNA expression in different types of thyroid lesions. Compared to benign tumors, miR-100, miR-125b, miR-138, and miR-768-3p were significantly downregulated in all malignant tumors, including HCTC [26]. Our results on miR-138 and miR-768-3p are in accordance with this study that reported that these miRNAs would be useful in diagnostic discrimination between benign Hürthle cell tumors and HCTC as the diagnostic accuracy of miR-138 and miR-768-3p for Hürthle cell neoplasms in their study was 98% [26]. Mitomo et al. reported that miR-138 is regulating the human telomerase reverse transcriptase (hTERT) resulting in progressive shortening of telomeres [32], suggesting that the role of miR-138 in cancer is biologically plausible.

In our study, there were no significant differences in expression of miR-885-5p between HCTC and normal thyroid tissue when all patients were analyzed. On the other hand, we have observed a slightly decreased miR-885-5p relative expression in tumor compared to normal tissue of patients with metastatic HCTC; however, in patients without metastases, the relative expression of miR-885-5p was significantly higher in HCTC compared to normal thyroid tissue, although all the levels were low. The highest relative expression levels of miR-885-5p were observed in the tumor tissue of HCTC patients without metastases and were significantly higher compared to patients with metastatic disease. Dettmer et al. found miRNA (miR-885-5p) to be strongly upregulated (from 4-fold to 191-fold) in 88% of patients with HCTC in comparison to benign tumors [17], while we only observed upregulation in nonmetastatic disease. So, miR-885-5p may serve as a diagnostic marker for HCTC; however, more studies are needed to elucidate its role in metastatic disease.

We found no significant differences in expression of miR-221 between HCTC and normal thyroid tissue when all patients were analyzed. Significant upregulation of miR-221 was observed in our study in HCTC compared to normal thyroid tissue only in patients without metastases, while in metastatic disease the relative expression in tumor tissue was lower, but it did not reach statistical significance. Furthermore, when the fold change of miRNA expression in tumor versus normal samples was compared between patients with and without metastatic disease, significant downregulation of miR-221 was observed in patients with a metastatic disease. Additionally, miR-183 was significantly downregulated in patients with metastases compared to patients without metastases. MiR-183 and especially miR-221 are often upregulated compared to normal tissue in different tumors, including thyroid cancer [17, 18]. Overexpressed miR-221 in papillary thyroid carcinomas regulates p27 protein levels and cell cycle [33]. However, different subtypes of HCTC can have different miRNA expression pattern, and both miR-183 and miR-221 showed loss of expression in poorly differentiated HCTC [18]. Our results are therefore in concordance with previous studies, as we only observed upregulation of miR-221 in tumor compared to normal tissue in nonmetastatic disease, but there was important downregulation of miR-183 and miR-221 in metastatic disease.

Different subtypes of HCTC can have different prognosis; therefore, it would be useful to determine specific miRNA expression profile that would also facilitate diagnosis for each of them. Previous studies about prognosis of patients with HCTC focused mostly on poorly differentiated tumors. Dettmer et al. [18] used PCR microarrays to study the expression of 768 miRNAs in 14 poorly differentiated, 13 oncocytic poorly differentiated, and 72 well differentiated thyroid carcinomas and 8 normal thyroid specimens. Several miRNAs were deregulated in poorly differentiated oncocytic tumors compared to normal tissue, including miR-221 and miR-885-5p. However, when poorly differentiated HCTCs were compared to well differentiated HCTCs, miR-125a-5p, miR-183-3p, miR-219-5p, miR-221, and miR-885-5p showed loss of expression [18]. In our study, only two cases fulfilled Turin criteria [24] for poorly differentiated thyroid carcinoma, making it difficult to compare miRNA expression. But we found no significant differences of relative expression of miRNA in patients with minimally invasive tumors in comparison to patients with widely invasive or poorly differentiated tumors.

Dettmer et al. [18] also observed that miR-23b was associated with tumor relapses, while miR-150 was associated with tumor specific death in patients with poorly differentiated HCTC [18]. Data about the predictive value of miR23-b and miR-150 were not published at the time when our study was planned. Because of the limited funds for our study, we did not analyze a wider range of miRNAs and, unfortunately, miR-150 and miR-23b were not included in our panel of tested miRNAs.

It has to be noted that comparison of miRNA studies can be difficult, as rarely the same comparisons are performed: the main differences are usually the type of samples used as controls (normal tissue samples may be from the same, matched, or unrelated patients) and methods used for the normalization of the results. Previous studies regarding thyroid carcinoma used different standards for normalization, making it difficult to directly compare the results. Among the studies most relevant for HCTC, one study used U18 for normalization of miRNA expression [24], while two used RNU44 and U6 [17, 18], suggesting that attention should be paid to normalization when comparing results.

Our study also faced limitations such as a small samples size and a limited set of included miRNAs. We have therefore made use of very strict inclusion criteria to decrease the heterogeneity among patients. Furthermore, all the patients were from a small and genetically homogenous population and were treated and followed up in the same institution. One important advantage of our study was also the use of paired normal and tumor tissue samples, as this could help uncover specific differences in miRNA expression. Moreover, we were the first to compare miRNA expressions in patients with and without metastatic disease. This allowed us to identify some miRNAs that are differentially expressed between the tumor and normal HCTC tissue and that could be further validated as biomarkers of metastatic disease in larger patient sets.

## 5. Conclusions

The most important finding of our study was that expression of miRNA (miR-183, miR-221, and miR-885-5p) in tumor tissue was associated with occurrence of distant metastases in patients with HCTC.

## Figures and Tables

**Table 1:** Clinical, demographic, and histopathological characteristics of patients with Hürthle cell thyroid carcinoma.

	Without metastases (*N* = 22)	With metastases (*N* = 17)	*p* value
Median age [years] (range)	52 (19–82)	63 (36–85)	0.006
Gender female [*N*] (%)	15 (68)	10 (59)	0.546
Median tumor diameter [cm] (range)	3 (1.8–6.5)	6.5 (3–18)	0.001
pT3 or pT4 tumor stage (%)	8 (36)	14 (82)	0.008
N1 or N2 stage (%)	1 (4)	3 (18)	0.300
M1 stage (%)	0 (0)	2 (12)	0.184
Poorly differentiated tumor, Turin criteria (%)	1 (4)	1 (6)	1.000
Widely invasive (%)	5 (22)	4 (24)	0.95
Minimally invasive (%)	17 (77)	11 (65)	0.39
Extensive vascular invasion (%)	4 (18)	5 (29)	0.46
Transcapsular invasion (%)	9 (41)	4 (24)	0.32
Recurrence (%)	0 (0)	15 (88)	—

**Table 2:** Relative expression of miRNAs in normal and tumor samples of all patients.

miRNA	Relative expression in normal tissue Median (25–75%)	Relative expression in tumor tissue Median (25–75%)	*p* value (Wilcoxon test for paired samples)
miR-138	1.54 (0.37–5.92)	0.15 (0.03–0.90) [1]	0.013
miR-183	0.02 (0.01–0.08) [3]	0.02 (0.01–0.13) [3]	0.357
miR-221	0.56 (0.26–1.95)	0.77 (0.20–4.08) [2]	0.645
miR-222	3.66 (1.76–8.23) [6]	4.00 (1.68–17.6) [6]	0.264
miR-768-3p	4.02 (1.64–9.76) [1]	1.72 (0.74–3.47) [2]	0.010
miR-885-5p	0.01 (0.00–0.06) [3]	0.02 (0.00–0.06) [1]	0.706

The amount of missing data is presented in brackets [ ].

**Table 3:** Relative expression of miRNAs in normal and tumor samples of patients without metastases.

miRNA	Relative expression in normal tissue Median (25–75%)	Relative expression in tumor tissue Median (25–75%)	*p* value (Wilcoxon test for paired samples)
miR-138	0.78 (0.08–2.65)	0.09 (0.01–1.40)	0.249
miR-183	0.02 (0.00–0.04)	0.03 (0.01–0.20)	0.067
miR-221	0.30 (0.15–0.60)	0.61 (0.14–5.79)	0.042
miR-222	3.43 (1.56–5.12)	4.18 (1.7–16.50)	0.123
miR-768-3p	3.00 (1.56–5.51)	1.80 (0.72–5.75)	0.101
miR-885-5p	0.01 (0.00–0.01) [1]	0.02 (0.00–0.07)	0.027

The amount of missing data is presented in brackets [ ].

**Table 4:** Relative expression of miRNAs in normal and tumor samples of patients with metastases.

miRNA	Relative expression in normal tissue Median (25–75%)	Relative expression in tumor tissue Median (25–75%)	*p* value (Wilcoxon test for paired samples)
miR-138	5.66 (1.35–42.5)	0.36 (0.12–1.19) [1]	0.030
miR-183	0.09 (0.02–0.4) [3]	0.02 (0–0.11) [3]	0.286
miR-221	2.4 (0.39–11.18)	0.77 (0.2–3) [2]	0.061
miR-222	10.12 (1.88–22.34) [6]	3.7 (1.04–27.52) [6]	0.790
miR-768-3p	7.08 (2.5–28.58) [1]	1.28 (0.76–2.53) [2]	0.048
miR-885-5p	0.04 (0.01–1.05) [2]	0.02 (0.01–0.08) [1]	0.074

The amount of missing data is presented in brackets [ ].

**Table 5:** Comparison of relative expressions of miRNA between patients without metastases and patients with metastases.

miRNA	All patients	Patients without metastases	Patients with metastases	*p* value Mann-Whitney *U* test
miR-138	0.13 (0.01–0.99) [1]	0.19 (0.02–1.68)	0.06 (0.01–0.64) [1]	0.261
miR-183	1.23 (0.57–5.75) [6]	1.54 (0.76–11.02)	0.63 (0.07–1.64) [6]	0.027
miR-221	1.06 (0.26–8.76) [2]	2.89 (0.47–22.20)	0.35 (0.07–1.83) [2]	0.019
miR-222	0.99 (0.45–5.63) [6]	1.02 (0.54–9.01)	0.68 (0.23–2.52) [6]	0.181
miR-768-3p	0.62 (0.19–1.06) [3]	0.66 (0.26–1.19)	0.42 (0.08–1.00) [3]	0.218
miR-885-5p	1.03 (0.20–5.32) [4]	2.84 (0.42–9.98) [1]	0.45 (0.02–1.25) [3]	0.024

The amount of missing data is presented in brackets [ ].

**Table 6:** Comparison of relative expressions of miRNA between patients with minimally invasive tumors and patients with widely invasive or poorly differentiated tumors.

miRNA	Minimally invasive tumors	Widely invasive/poorly differentiated tumors	*p* value (Mann-Whitney *U* test)
miR-138	0.12 (0.01–0.71) [1]	0.23 (0.05–1.64)	0.394
miR-183	1.06 (0.57–3.95) [3]	1.35 (0.24–11.21) [3]	0.867
miR-221	0.59 (0.25–4.6) [1]	3.23 (0.25–12.24) [1]	0.356
miR-222	0.98 (0.47–6.73) [4]	1.97 (0.28–5.09) [2]	0.872
miR-768-3p	0.42 (0.18–1.01) [3]	0.64 (0.21–1.18)	0.932
miR-885-5p	1.13 (0.11–5.39) [3]	0.97 (0.45–5.91) [1]	0.827

The amount of missing data is presented in brackets [ ].
